# The effect of spike time dependent plasticity on activity patterns in the basal ganglia

**DOI:** 10.1186/1471-2202-12-S1-P351

**Published:** 2011-07-18

**Authors:** Marcel AJ Lourens, Jasmine A Nirody, Hil GE Meijer, Tjitske Heida, Stephan A van Gils

**Affiliations:** 1 Department of Applied Mathematics, University of Twente, Enschede, 7500 AE, The Netherlands; 2 New York Medical College, Valhalla, NY 10595, USA; 3 Department of Electrical Engineering, University of Twente, Enschede, 7500 AE, The Netherlands

## Background

Pathophysiology of Parkinson's disease (PD) is characterized by increased firing rates of cells in the basal ganglia, a tendency toward bursting and abnormal synchronization in cells of subthalamic nucleus (STN) and globus pallidus pars externa (GPe) [1]. In advanced PD, deep brain stimulation (DBS) can be used to disrupt this pathological activity. The standard protocol for DBS is continuous high frequency stimulation of target cells, such as STN. It has been proposed that short-duration stimulation protocols may also disrupt the pathological activity [2]. The mechanism underlying this protocol is supposedly synaptic plasticity. The goal of this study is to investigate, with a biophysically plausible model, the role of synaptic plasticity in stabilizing firing patterns in the basal ganglia.

## Method

We use a STN-GPe network model consisting of 8-20 STN and GPe cells connected to each other via a sparse structured architecture [3]. The dynamics of each cell is described by a single-compartment conductance-based model. We change the weights of the excitatory projections from STN to GPe with a pre-post spike-timing dependent plasticity (STDP) rule. We apply DBS as a train of positive current pulses injected directly into each STN cell.

## Results

Depending on both cell and synaptic parameters, the network model without STDP displays a variety of activity patterns, including synchronous, correlated dynamics and irregular firing. In the model with STDP, the nearly clustered activity patterns disappears and we observe only irregular firing or synchronized activity. Application of DBS replaces the clustered activity by high frequency firing. Switching DBS off, the network without STDP returns to a pathological activity pattern. In contrast, with STDP the temporary application of DBS changes the connectivity such that the network does not fall back to the clustered activity.

## Discussion

Synaptic plasticity is the nervous system’s way to learn and adapt to sensory inputs, and this adaptation can strengthen both desired and pathological states. STDP pushes network dynamics to irregular (health) or synchronized (pathological) state, depending on parameter values. Moreover, our results suggest that DBS can use STDP to teach the network to fire in a less pathological manner. This teaching ability of DBS can be exploited to develop more efficient stimulation protocols.
